# Supplementary material for: Invasion of vaginal epithelial cells by uropathogenic Escherichia coli
Source: Nat Commun. 2020 Jun 4;11:2803. doi: 10.1038/s41467-020-16627-5 (PMC7272400; doi:10.1038/s41467-020-16627-5)
Supplement: Supplementary file 1 — Supplementary Information [file 41467_2020_16627_MOESM1_ESM.pdf]

Supplementary Information

Brannon et al.

Supplementary Figure 1

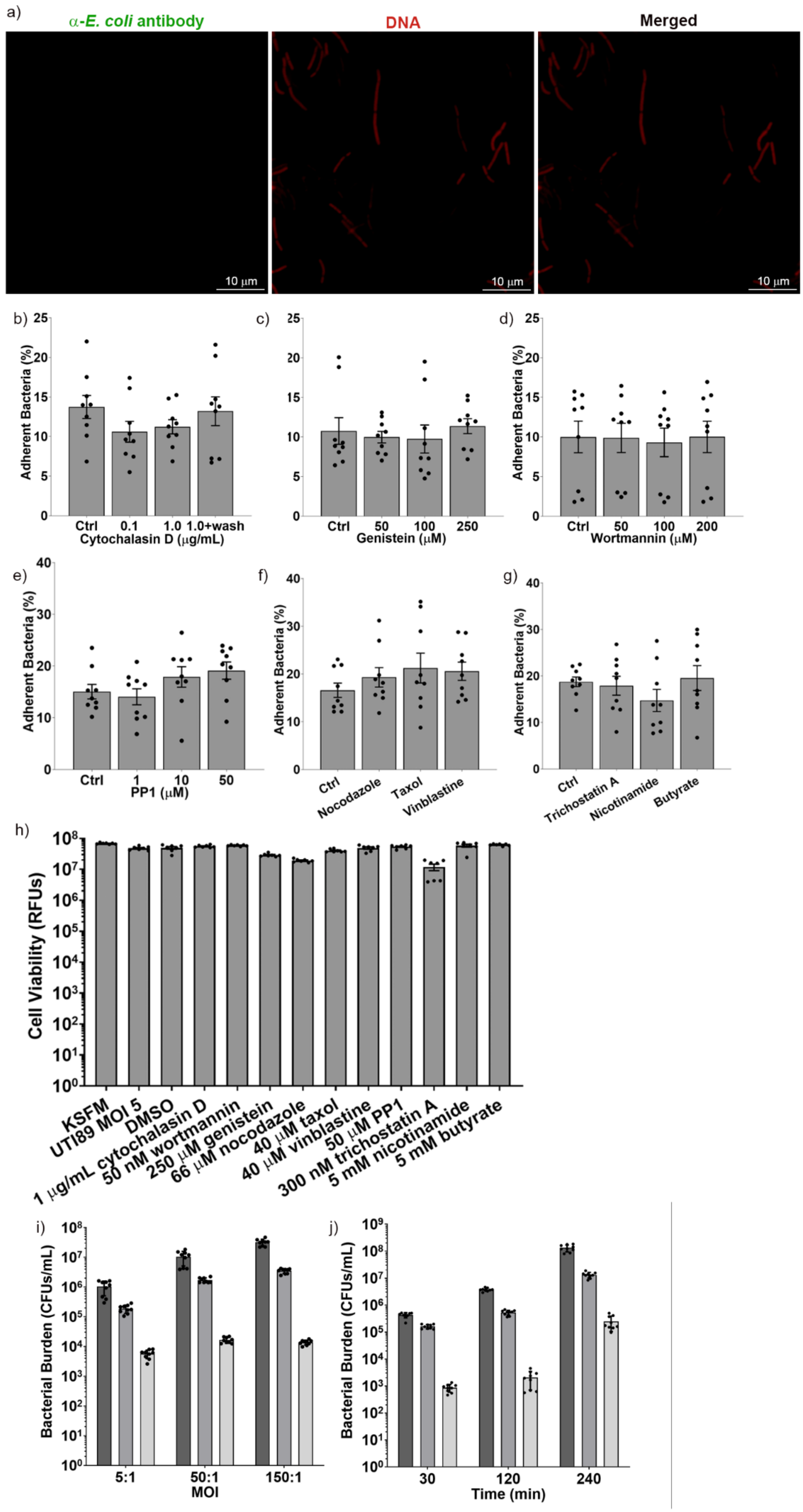

Supplementary Figure 1. Tested inhibitors do not significantly impact UPEC adherence to or viability of VK2 E6/E7 Cells. a, Representative image (n = 3) of immunofluorescence microscopy of *L. crispatus* ATCC 33197 stained with the α-*E. coli* antibody (green) and with ToPro-3 as a DNA counterstain (red). The α-*E. coli* antibody used is specific for *E. coli*. **b-g,** Adherence and invasion assay was performed for two hours at a MOI of 5 with UTI89. Data are representative of the adherent population of bacteria corresponding to the invasion experiment in Fig. 2d-i with the mean of nine independent experiments with error bars representing s.e.m. **h,** Cell viability was determined with PrestoBlue (ThermoFisher Scientific) according to manufactures instructions. Data are representative of the mean of seven independent experiments with error bars representing s.e.m. For statistical analysis Kruskal-Wallis with two-sided Dunn's post-hoc test used and no statistically significant difference ($P > 0.05$) was found between treatments and respective controls. **i, j,** Adherence and invasion assay shown as CFUs/mL corresponding to the data in Fig. **i,** 1a and **j,** 1b. The percentage of adherent and intracellular bacteria decreases as *E. coli* grows within the extracellular media within wells. Data are representative of the mean of nine independent experiments with error bars representing standard deviation.

Supplementary Table 1. Bacterial strains and plasmids used in this study.

E. coli **strains**	**Details**	**Source or Reference**
UTI89	A prototypical wild-type strain isolated from a case of cystitis	25
Δ*fimA-H*	UTI89 with the *fim* operon deleted	25
Vanderbilt Urinary Tract Isolates (VUTI)	Isolates were obtained through the microVU biobank	42
VUTI 126	Isolated from a woman with cystitis	42
VUTI 134	Isolated from a woman with cystitis	42
VUTI 175	Isolated from a woman with cystitis	42
VUTI 165	Isolated from a woman with rUTI	42
VUTI 173	Isolated from a woman with rUTI	42
VUTI 234	Isolated from a woman with rUTI	42
VUTI 170	Isolated from a woman with ASB	42
VUTI 205	Isolated from a woman with bacteremia	42
VUTI 385	Isolated from a woman with bacteremia	42
VUTI 115	Isolated from a woman with pyelonephritis	42
**Other bacteria**	**Details**	**Reference**
L. crispatus ATCC 33197	Obtained from American Type Culture Collection (ATCC)	71
**Plasmid**	**Details**	**Reference**
pCom_GFP	Plasmid constitutively expressing *gfp*	25
