# Supplementary material for: Invasion of vaginal epithelial cells by uropathogenic Escherichia coli
Source: Nat Commun. 2020 Jun 4;11:2803. doi: 10.1038/s41467-020-16627-5 (PMC7272400; doi:10.1038/s41467-020-16627-5)
Supplement: Supplementary file 3 — Description of Additional Supplementary Files [file 41467_2020_16627_MOESM3_ESM.pdf]

Description of Additional Supplementary Files

File Name: Supplementary Movie 1
Description: UPEC Invasion of Murine VECs During Acute UTI. Representative Z-stack images compiled into a video of vaginal epithelium colonized by UPEC. Immunofluorescence was performed with α-E. coli antibody (green), rWGA (red), and ToPro-3 (blue) staining. The video displays vaginal intracellular communities within VECs.

File Name: Supplementary Movie 2
Description: UPEC Invasion of Murine VECs in a Model of Chronic UTI. Representative Z-stack images compiled into a video of vaginal epithelium colonized by UPEC. Immunofluorescence was performed with α-E. coli antibody (green), rWGA (red), and ToPro-3 (blue) staining. The video displays vaginal intracellular communities within VECs.

File Name: Supplementary Movie 3
Description: Three-dimensional Surface Projection of a VEC Containing a Vaginal Intracellular Community Isolated from a Patient with rUTI. Z-stack images were analyzed with Imaris software to generate a three-dimensional surface projection of a human VEC with invading UPEC. Immunofluorescence microscopy with α-E. coli (green), cytokeratin 13 (red), and α-uroplakin III (yellow), and ToPro-3 (blue) staining.
