# Supplementary material for: Invasion of vaginal epithelial cells by uropathogenic Escherichia coli
Source: Nat Commun. 2020 Jun 4;11:2803. doi: 10.1038/s41467-020-16627-5 (PMC7272400; doi:10.1038/s41467-020-16627-5)
Supplement: Supplementary file 7 — Reporting Summary [file 41467_2020_16627_MOESM7_ESM.pdf]

nature research

Corresponding author(s): Maria Hadjifrangiskou and John R. Brannon

Last updated by author(s): Jun 27, 2019

Reporting Summary

Nature Research wishes to improve the reproducibility of the work that we publish. This form provides structure for consistency and transparency in reporting. For further information on Nature Research policies, see Authors & Referees and the Editorial Policy Checklist .

Statistics

For all statistical analyses, confirm that the following items are present in the figure legend, table legend, main text, or Methods section.

n/a	Confirmed	
☐	☒	The exact sample size (*n*) for each experimental group/condition, given as a discrete number and unit of measurement
☐	☒	A statement on whether measurements were taken from distinct samples or whether the same sample was measured repeatedly
☐	☒	The statistical test(s) used AND whether they are one- or two-sided<br>*Only common tests should be described solely by name; describe more complex techniques in the Methods section.*
☐	☒	A description of all covariates tested
☐	☒	A description of any assumptions or corrections, such as tests of normality and adjustment for multiple comparisons
☐	☒	A full description of the statistical parameters including central tendency (e.g. means) or other basic estimates (e.g. regression coefficient) AND variation (e.g. standard deviation) or associated estimates of uncertainty (e.g. confidence intervals)
☐	☒	For null hypothesis testing, the test statistic (e.g. *F*, *t*, *r*) with confidence intervals, effect sizes, degrees of freedom and *P* value noted<br>*Give P values as exact values whenever suitable.*
☒	☐	For Bayesian analysis, information on the choice of priors and Markov chain Monte Carlo settings
☒	☐	For hierarchical and complex designs, identification of the appropriate level for tests and full reporting of outcomes
☒	☐	Estimates of effect sizes (e.g. Cohen's *d*, Pearson's *r*), indicating how they were calculated

Our web collection on statistics for biologists contains articles on many of the points above.

Software and code

Policy information about availability of computer code

Data collection	Microscopy images were collected with Zen (Ziess International, Oberkochen Germany) software both of which are publicly available.
Data analysis	Images were analyzed with Zen version 2.6 blue edition (Zeiss International, Oberkochen Germany) and Imaris (Bitplane, Zürich Switzerland) softwares where indicated in the manuscript. Images were cropped in Photoshop CC version 21.1.2 (Adobe, San Jose, CA) for their inclusion in figures. GraphPad Prism version 8.3 was used for all statistical analyses performed using the most appropriate test as designated within the figure legends. All software use in this study is publicly available.

For manuscripts utilizing custom algorithms or software that are central to the research but not yet described in published literature, software must be made available to editors/reviewers. We strongly encourage code deposition in a community repository (e.g. GitHub). See the Nature Research guidelines for submitting code & software for further information.

Data

Policy information about availability of data

All manuscripts must include a data availability statement. This statement should provide the following information, where applicable:
- Accession codes, unique identifiers, or web links for publicly available datasets
- A list of figures that have associated raw data
- A description of any restrictions on data availability

The authors declare that all data supporting the findings of this study are available within this manuscript and its supplementary information files.

Field-specific reporting

Please select the one below that is the best fit for your research. If you are not sure, read the appropriate sections before making your selection.

- [x] Life sciences
- [ ] Behavioural & social sciences
- [ ] Ecological, evolutionary & environmental sciences

For a reference copy of the document with all sections, see nature.com/documents/nr-reporting-summary-flat.pdf

Life sciences study design

All studies must disclose on these points even when the disclosure is negative.

Sample size	For bacterial based assays, we did not use statistical methods to determine sample size because the work reported contained samples with bacteria on the magnitude of 106 bacterial cells. For murine in vivo fitness studies, power analysis software at http://statisticalsolutions.net/pss_calc.php was used to calculate the number of mice required to obtain a statistically significant measure of differences corresponding to 75% of the mean between groups, assuming 20% of the mean. For the preliminary pilot study on searching for vaginal cell invasion in women, the number of vaginal samples was limited by the number of consenting participants.
Data exclusions	No data were excluded.
Replication	The results were reproducible amongst multiple biological replicates . The number of replicates are indicated within each figure.
Randomization	Bacterial strains selected from the microVU bank for testing were selected based upon availability and relevant criteria specified within the methods section, including disease and patient sex. After inoculation mice were separated into cages based upon urine titers described in the methods section. Tissue for microscopy was randomly selected from the cohorts.
Blinding	Blinding was not necessary for these experiments as result were binary (cell invasion or not). For the experiments in this study the researchers were not blinded and relied upon the reproducibility between biological replicates and models. The researcher was blinded to mouse cohort, patient information and questionnaire for performing microscopy on microscopy of mouse tissue and patient vaginal samples.

Reporting for specific materials, systems and methods

We require information from authors about some types of materials, experimental systems and methods used in many studies. Here, indicate whether each material, system or method listed is relevant to your study. If you are not sure if a list item applies to your research, read the appropriate section before selecting a response.

Materials & experimental systems

n/a	Involved in the study	
☐	☒	Antibodies
☐	☒	Eukaryotic cell lines
☒	☐	Palaeontology
☐	☒	Animals and other organisms
☐	☒	Human research participants
☒	☐	Clinical data

Methods

n/a	Involved in the study	
☒	☐	ChIP-seq
☒	☐	Flow cytometry
☒	☐	MRI-based neuroimaging

Antibodies

Antibodies used	Antibodies in this study include rabbit anti-E. coli (US Biologicals, 3500-06, Lot L8102865), mouse anti-uroplakin III (Abcam, ab78196, SFI-1), goat anti-cytokeratin 13 (Abcam, ab79279), donkey anti-rabbit IgG Alex Fluor 488 conjugated (ThermoFisher Scientific, A21206), donkey anti-goat IgG Alex Fluor 546 conjugated (ThermoFisher Scientific, A11056), donkey anti-mouse IgG Alexa Fluor 594 conjugated (ThermoFisher Scientific, A21203), and donkey anti-rabbit IgG Alexa Fluor 647 conjugated (ThermoFisher Scientific, A31573).
Validation	Primaries were validated by the individual suppliers for validation for immuno-staining purposes as stated in the suppliers certificate of analysis provided on their website. As per suppliers website, the goat anti-cytokeratin 13 antibody was raised against synthetic peptide to the C-terminal residues 444-458 and validated for specificity for human cytrokeratin 13 using human lung tissue. As per the supplier, the mouse monoclonal anti-uroplakin III antibody was raised against synthetic peptide corresponding to human uroplakin III and validated with urothelial tissue as reactive with mouse and human uroplakin III. The rabbit anti-E. coli antibody was confirmed to be none reactive with antigens of the vaginal microbiota as presented within Supplementary Figure 1.

Eukaryotic cell lines

Policy information about cell lines

Cell line source(s)	VK2 E6/E7 cells were directly obtained from American Type Culture Collection nonprofit organization. 5637 (ATCC HTB-9) cells were obtained from the laboratory of Dr. Andries Zijlstra, who confirmed the authentication and lack of mycoplasma contamination of the cell line.
Authentication	These cells were not authenticated as they were obtained directly from the authenticating bio-repository.
Mycoplasma contamination	Cell lines were not tested for contamination of mycoplasma.
Commonly misidentified lines (See ICLAC register)	These cell lines are not commonly misidentified.

Animals and other organisms

Policy information about studies involving animals; ARRIVE guidelines recommended for reporting animal research

Laboratory animals	This study used 7-8 weeks old C3H/HeN female mice (Envigo). Mouse bedding was changed at least weekly by the facility and kept on a 12-hour dark and 12-hour light cycle.
Wild animals	This study did not involve wild animals.
Field-collected samples	This study did not use field collected samples
Ethics oversight	All mouse experiments were approved by the Vanderbilt University Medical Center Institutional Animal Care and Use Committee (IACUC) (protocol numbers M/12/191, M1500017-01, and M1800101-00). All experiments were conducted in accordance with the guidelines of the National Institute of Health and IACUC at VU MC.

Note that full information on the approval of the study protocol must also be provided in the manuscript.

Human research participants

Policy information about studies involving human research participants

Population characteristics	Women of 18 years of age or older with a history of prior UTI or rUTI, defined as 3 or more UTIs in the past 12 months, were enrolled in this study. Women with urinary tract or pelvic floor abnormalities or major medical conditions that in the opinion of the investigator would place the individual or study at risk were excluded.
Recruitment	Participants were enrolled with their informed consent during their visit to the Urology Clinic, within The Vanderbilt Clinic at the Vanderbilt University Medical Center. Participants self-reported in a questionnaire about relevant clinical data. Questionnaire information was not used in data interpretation and as such does not contribute to self-selection bias.
Ethics oversight	The Vanderbilt University Medical Center Human Research Protections Program approved clinical sample collection by internal review board (#180973) oversight.

Note that full information on the approval of the study protocol must also be provided in the manuscript.
